# Low myelin-related values in the fornix and thalamus of 7 Tesla MRI of major depressive disorder patients

**DOI:** 10.3389/fnmol.2023.1214738

**Published:** 2023-08-10

**Authors:** Jeong-Min Shim, Seo-Eun Cho, Chang-Ki Kang, Seung-Gul Kang

**Affiliations:** ^1^Department of Nano Science and Technology, Gachon University Graduate School, Seongnam, Republic of Korea; ^2^Department of Psychiatry, Gil Medical Center, Gachon University College of Medicine, Incheon, Republic of Korea; ^3^Neuroscience Research Institute, Gachon University, Incheon, Republic of Korea; ^4^Department of Radiological Science, College of Health Science, Gachon University, Incheon, Republic of Korea

**Keywords:** major depressive disorder, myelin-related map, relaxation times, ratio mapping, ME-MP2RAGE, 7 T MRI

## Abstract

**Introduction:**

Abnormalities in myelin are believed to be one of the important causes of major depressive disorder, and it is becoming important to more accurately quantify myelin in *in vivo* magnetic resonance imaging of major depressive disorder patients. We aimed to investigate the difference in myelin concentration in the white matter and subcortical areas using new quantitative myelin-related maps of high-resolution 7 Tesla (7 T) magnetic resonance imaging between patients with major depressive disorder and healthy controls.

**Methods:**

Myelin-related comparisons of the white matter and nearby subcortical regions were conducted between healthy controls (*n* = 36) and patients with major depressive disorder (*n* = 34). Smoothed quantitative ratio (sq-Ratio) myelin-related maps were created using the multi-echo magnetization-prepared two rapid gradient echoes (ME-MP2RAGE) sequence of the T1 and T2* images of 7 T magnetic resonance imaging. Differences in the myelin-related values of the regions of interest between the two groups were analyzed using a two-sample t-test, and multiple comparison corrections were performed using the false discovery rate.

**Results:**

The average sq-Ratio myelin-related values were 2.62% higher in the white matter and 2.26% higher in the subcortical regions of the healthy controls group than in the major depressive disorder group. In the group analysis of the healthy control and major depressive disorder groups, the sq-Ratio myelin-related values were significantly different in the fornix area of the white matter (false discovery rate-corrected *p* = 0.012). In addition, significant differences were observed in both the left (false discovery rate-corrected *p* = 0.04) and right thalamus (false discovery rate-corrected *p* = 0.040) among the subcortical regions.

**Discussion:**

The average sq-ratio myelin-related value and sq-ratio myelin-related values in the fornix of the white matter and both thalami were higher in the healthy controls group than in the major depressive disorder group. We look forward to replicating our findings in other populations using larger sample sizes.

## 1. Introduction

Major depressive disorder (MDD) is a psychiatric disorder and serious illness that significantly impairs quality of life and is the most common cause of suicide (Cho et al., 2016). In 2008, the World Health Organization ranked MDD as the third most common global disease and predicted that it would become the most common in 2030 (World Health Organization, 2008). The causes of MDD are abnormalities in neurotransmitters, such as the monoamine hypothesis; hypothalamic–pituitary–adrenal axis changes; abnormalities in neuronal networks; genetic factors; stressors; and experiences such as childhood abuse; however, the biological mechanisms of depression are still not completely understood.

Recently, there has been increased interest in the myelin sheath and oligodendrocyte lineage cells, and their role in the central nervous system (CNS; Zhou et al., 2021). Oligodendrocyte lineage cells is the collective term for oligodendrocyte progenitor cells and mature oligodendrocytes, which form myelin sheaths around axons and are known to physically and metabolically support axons and mediate the process of neuroplasticity (Zhou et al., 2021). In this manner, oligodendrocyte lineage cells form the white matter, which is a major component of the CNS. Myelin wraps around the axons of the neurons to maintain the efficiency of brain function, maintain nerve fiber integrity, and accelerate the propagation of action potentials (Salzer, 2015). The role of myelin in several neuropsychiatric disorders, such as multiple sclerosis, acute disseminated encephalomyelitis, depression, and schizophrenia, has been studied (Van der Knaap and Valk, 2005). Disordered synaptic transmission may cause abnormal brain development and major psychiatric disorders, such as MDD and schizophrenia (Davis et al., 2003). Myelin has also been implicated in the pathogenesis of depression (Zhou et al., 2021), and numerous studies have implicated myelination as a critical process affecting neuronal connectivity (Davis et al., 2003; Mighdoll et al., 2015; Moura et al., 2021).

In MDD, brain imaging studies have used techniques such as structural abnormalities, functional connectivity, and diffusion tensor imaging depending on the objectives and hypotheses of the study. Recent brain imaging studies have shown that abnormalities in white matter hyperintensities and myelin integrity start to change in the prefrontal areas in the early stages of depression (Cole et al., 2012). In previous studies, white matter tract regions in various brain regions decreased significantly (Taylor et al., 2004; Aston et al., 2005; Bae et al., 2006; Benedetti et al., 2011; Zhang et al., 2013; Bhatia et al., 2018; Williams et al., 2019). Samples from patients have shown a reduction in myelin content and axon numbers in various brain areas (Regenold et al., 2007; Tham et al., 2011). This suggests that individuals with depression may have differences in the structure and function of myelin in the brain compared with those without depression, particularly in the prefrontal cortex, which is involved in emotional regulation, decision-making, and other cognitive processes.

Myelin abnormalities are an important cause of depression, and it is important to accurately quantify myelin using *in vivo* magnetic resonance imaging (MRI) for more accurate research into the pathogenesis of depression. In a previous study, we introduced a myelin-related mapping technique to obtain quantitative maps using 7 Tesla MRI (Shim et al., 2022). In this study, the term “quantitative maps” refers to maps derived from quantitative R1 and T2* images, which are not indicative of the anatomical content of myelin, but rather obtained through imaging parameters that are independent and quantitative in MRI. This technique improves the myelin contrast between the myelinated and unmyelinated areas by dividing the R1 and T2* images in which the received bias field is removed. This enables the quantification of myelin-related density using R1 and T2* images instead of weighted images with varying signal intensity values according to the selected MR parameters. In addition, this technique uses a signal MR pulse sequence, such as multi-echo magnetization-prepared by two rapid gradient-echo (ME-MP2RAGE) sequences, which is not necessary for further image registration (Metere et al., 2017). Two or more MR pulse sequences are used to obtain R1 and T2* images, requiring a long acquisition time and further correction of the motion artifacts. Therefore, this technique can provide objective and accurate quantification of myelin using high-resolution quantitative images in a relatively short scan time (Shim et al., 2022), and can be applied to the study of various neuropsychiatric diseases related to demyelination, including depression.

Several quantitative MRI (qMRI) methods have been developed and used to measure myelin-related properties of white matter in patients with MDD (Sacchet and Gotlib, 2017). Sacchet et al. measured *in vivo* myelin concentration through R1 (1/T1), compared myelin concentration between patients with MDD and healthy controls, and revealed that patients with MDD had lower levels of myelin at the whole-brain level and in the nucleus accumbens (Sacchet and Gotlib, 2017). Hou et al. compared the myelin levels between patients with MDD and controls using an inhomogeneous magnetization transfer method, which is a surrogate measure of myelin content, and showed myelin impairment in the fornix, left anterior limb of the internal capsule, and left sagittal striatum (Hou et al., 2021). We aimed to determine whether myelin concentration in the white matter and subcortical areas differs between patients with MDD and normal controls using 7 T high-resolution MRI.

## 2. Materials and methods

### 2.1. Subjects

Thirty-four patients with MDD and 36 healthy controls (HCs) were included in the study after providing written informed consent. Age, sex, and duration of education were matched between the two groups. The study was approved by the Institutional Review Board of Gil Medical Center (IRB No. GDIRB2018-005 and GDIRB2020-207). One board-certified psychiatrist (SGK) interviewed all the participants and assessed their eligibility for the study using a structured clinical interview based on the fifth edition of the Diagnostic and Statistical Manual of Mental Disorders (DSM-5) (SCID-5; First et al., 2016). Patients meeting the DSM-5 diagnostic criteria for MDD were included in the MDD group.

The common exclusion criteria for MDD and HC were as follows: age < 19 or > 65 years; left-handed use in the Edinburgh Handedness Test (Oldfield, 1971); current serious suicide risk; previous abnormal findings on brain imaging; contraindications to MRI (e.g., metals in the body); pregnancy or lactation; major or unstable medical and neurological disorders within the past year; history of head trauma; substance use disorder within the past year; intellectual disability; personality disorder; and neurocognitive disorders. Additional exclusion criteria for MDDs were comorbidities of major psychiatric disorders (i.e., schizophrenia spectrum and other psychotic disorders, obsessive–compulsive and related disorders, substance-related and addictive disorders, major anxiety disorders, and disruptive, impulse control, and conduct disorders). Further exclusion criteria for the HCs were as follows: Hamilton Depression Rating Scale 17 items (HDRS-17) total score > 6; psychiatric history; history of taking psychotropic medications; and first-degree relatives with schizophrenia, MDD, or bipolar disorder.

Depression severity was quantified using the HDRS-17 (Yi et al., 2005), Clinical Global Impression of Severity (CGI-S) (Busner and Targum, 2007), and Beck Depression Inventory (BDI) (Rhee et al., 1995) at baseline and on the MRI scanning date. Based on the HDRS-17 score, depression severity was classified as severe (≥25), moderate (18–24), mild (7–17), and no depression (0–6). We assessed depressive symptoms on the same day as the MRI scan.

### 2.2. Data acquisition

Brain images were acquired with an eight-channel phased array coil using a 7 T MRI system (Magnetom, Siemens, Erlangen, Germany). A prototype multi-echo magnetization-prepared two rapid gradient-echo (ME-MP2RAGE) sequence was used (Metere et al., 2017), and sagittal images were acquired using the following parameters: repetition time (TR) = 8,000 ms; four echo times (TEs) = 3.46, 7.28, 11.1, and 14.92 ms; two inversion times (TIs) = 1000/3200 ms; flip angle = 4°; field of view (FOV) = 166 × 166 × 135.2 mm^3^ with nominal isotropic resolutions of 0.65 mm; matrix size = 256 × 256; 208 slices along the right–left axis; bandwidth = 280 Hz/px; bipolar readout; generalized auto-calibrating partially parallel acquisitions (GRAPPA) with accelerating factor = 3 (50 reference lines); and 7/8 and 6/8 partial Fourier factors along the phase-encoding and slice-encoding directions, respectively, yielding an acquisition time (TA) = 14 min 16 s.

### 2.3. Image processing to acquire individual sq-ratio myelin-related maps

Images acquired from the ME-MP2RAGE sequence were used to reconstruct the T1 and T2* images. The T1 images were generated immediately after scanning through the reconstruction process provided by the sequence, but the R2* (= 1/T2*) images were reconstructed using the Fit T2 or T2 Star MRI data program^1^ with multiple echo images obtained after the second inversion radiofrequency pulse.

To generate myelin-related maps, T1 and R2* images were normalized using a voxel size of 0.5 mm to the standard space and the Montreal Neurological Institute (MNI) 152 template using statistical parametric mapping (SPM) 12 (Ashburner et al., 2020). T2* maps were generated from normalized R2* maps, and the T1 and T2* ranges were set to 700–4,000 ms and 1–60 ms (Metere et al., 2017), respectively. Quantitative ratio (q-Ratio) myelin-related maps were generated by dividing the R1 images by the T2* images (Shim et al., 2022).

**Figure 1:**
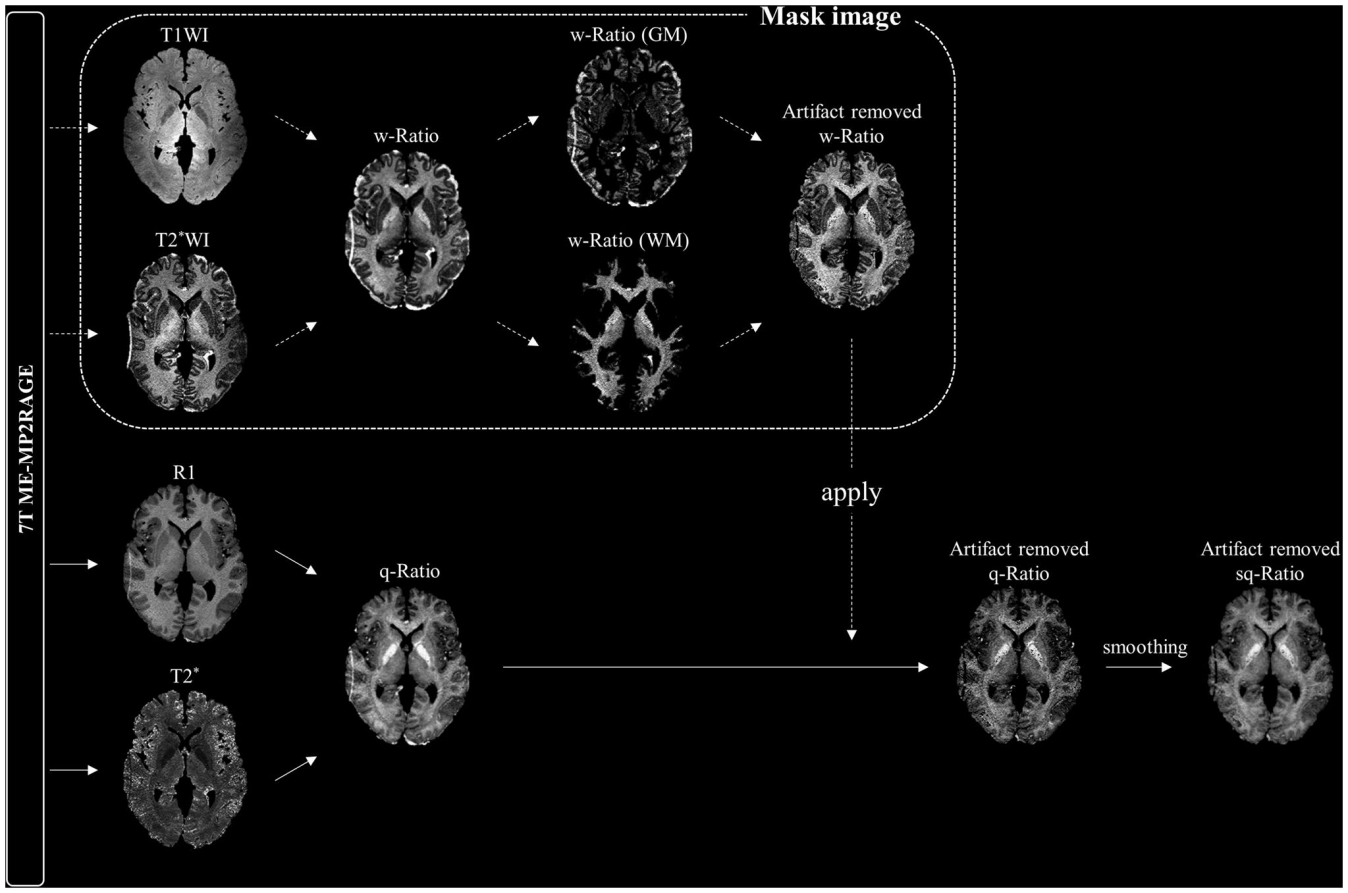
Schematic diagram of smoothed quantitative ratio (sq-Ratio) myelin-related mapping. For the mask image, we first created a weighted ratio (w-Ratio) myelin-related map by dividing the T1-weighted image (T1WI) and T2*-weighted image (T2*WI) obtained from the ME-MP2RAGE sequence, and then applied the quartile method to create an artifact-removed w-Ratio map. We applied this mask image to the q-ratio myelin-related map (q-ratio) to create an artifact-free map, which was then smoothed to 1 mm. GM, gray matter; WM, white matter.

To remove artifacts around the outer boundary of the brain images, which could make the analysis of myelin-related values difficult, we used weighted ratio (w-Ratio) images in which T2*-weighted (T2*W) images were divided from T1-weighted (T1W) images, providing a better contrast between white and gray matter (Shim et al., 2022). During preprocessing, the T1W and T2*W images obtained using the ME-MP2RAGE sequence were normalized to 0.5 mm on the MNI 152 template in the same manner as the T1 and R2* images. The resulting w-Ratio myelin-related images were generated by dividing the normalized T2*W image by the normalized T1W image (Figure 1). The standard T1 image provided by the SPM was segmented and divided into gray and white matter, which were used as mask images to reconstruct the w-ratio myelin-related images. Furthermore, the artifacts in the w-Ratio myelin-related images were removed after identifying outliers using the quartile method (Rousseeuw and Hubert, 2011).

Finally, the artifact-removed w-Ratio myelin-related image was used as a mask image to create a q-Ratio myelin-related image. The individual images were then smoothed with an isotropic three-dimensional Gaussian kernel of 1 mm full width at half maximum to obtain smoothed q-Ratio (sq-Ratio) myelin-related maps.

### 2.4. Group analysis and statistical analysis

We analyzed the myelin-related differences in the normal HCs and MDD groups, focusing primarily on the white matter, where myelin is predominantly located, as well as the nearby subcortical areas. We used the Johns Hopkins University (JHU) ICBM-DTI-81 white matter label atlas to analyze the white matter (Mori et al., 2008). To observe myelin in the subcortical and cerebellar areas adjacent to the white matter (Zhang F. F. et al., 2018), which are considered important in the recent onset mechanism of depression, we used the Automated Anatomical Labeling Atlas 2 (AAL2; Rolls et al., 2015). Both the JHU ICBM-DTI-81 white matter labels atlas and AAL2 were normalized to 0.5 mm, using Matlab 2018b (The MathWorks Inc., Natick, MA).

Differences in sq-Ratio myelin-related values between the MDD and HC groups in 50 white matter regions of interest (ROIs) and 40 subcortical ROIs were analyzed using Welch’s t-test, and multiple comparison corrections were performed using the false discovery rate (FDR; Benjamini and Hochberg, 1995). To further examine the impact of medication in the MDD group, we divided it into those taking and those not taking antidepressants, and used the same statistical methods to compare the two groups by medication status. The statistical threshold was set at *p* < 0.05. In addition, in the MDD group, we performed a partial correlation analysis (*p* < 0.05, two-tailed) to determine the statistical association of sq-Ratio myelin-related values (mean values in the subcortex, white matter, and regions that were significant when comparing the MDD and HC groups) with key clinical variables such as depression severity and duration of illness (Chen et al., 2022). All statistical analyses were performed using SPSS ver. 25 (IBM Corp., Armonk, NY) and Jamovi ver. 2.2.5 (The Jamovi Project [2021]).

## 3. Results

The demographic and clinical characteristics of the MDD and HC groups and their comparisons are shown in Table 1. There were no significant differences in age, sex ratio, or duration of education between the two groups. As expected, the MDD patients had significantly more severe depressive symptoms than the HC group, as measured using the HDRS and BDI (*p* < 0.001; Table 1). Based on HDRS scores, the severity of depression in the MDD group was severe, moderate, and mild in 2 (6%), 17 (50%), and 13 patients (38%), respectively. In addition, 28 patients in the MDD group were taking antidepressants, 14 of whom were taking more than one type thereof, and the antidepressants were escitalopram (*n* = 10), trazodon (*n* = 8), bupropion (*n* = 5), desvenlafaxine (*n* = 4), vortioxetine (*n* = 3), fluoxetine (*n* = 3), mirtazapine (*n* = 3), paroxetine (*n* = 3), milnacipran (*n* = 2), setraline (*n* = 1), agomelatine (*n* = 1), and imipramine (*n* = 1). Furthermore, in the MDD group, the average duration of depression was 5.53 years, and 11 patients had history of a previous suicide attempt. The BHS, CGI, and Scale for Suicide Ideation (SSI) scores were higher in patients with MDD than in the HCs (*p* < 0.001; Table 1).

**Table 1:** Demographic and clinical characteristics of the participants.

Clinical variables	MDD (*n* = 34)	HC (*n* = 36)	Statistics
Age at scan, years (mean ± SD)	35.3 ± 13.9	35.3 ± 12.4	*t* = 0.01, *p* = 0.990^a^
Sex (male:female)	10:24	10:26	*χ*^2^ = 0.02, *p* = 0.880^b^
Education, years (mean ± SD)	14.1 ± 2.4	14.8 ± 1.8	*t* = 1.36, *p* = 0.179^a^
Duration of illness, years (mean ± SD)	5.7 ± 5.3	N/A	N/A
Clinical scales at MRI scanning			
HDRS-17 score (mean ± SD)	16.4 ± 6.2	2.4 ± 2.3	*t* = −12.51, *p* < 0.001^a^
BDI score (mean ± SD)	27.1 ± 13.2	3.3 ± 3.6	*t* = −10.17, *p* < 0.001^a^
BHS score (mean ± SD)	11.7 ± 5.6	2.1 ± 1.4	*t* = −9.76, *p* < 0.001^a^
CGI-S score (mean ± SD)	4.2 ± 1.2	1.0 ± 0.1	*t* = −15.58, *p* < 0.001^a^
SSI score (mean ± SD)	15.3 ± 8.2	0.9 ± 1.5	*t* = −10.05, *p* < 0.001^a^

a Student’s *t*-test; ^b^Chi-square test.

Data are presented as means ± standard deviation or number (percentage), unless otherwise indicated. BDI, Beck Depression Inventory; BHS, Beck Hopelessness Scale; CGI-S, Clinical Global Impression Severity Scale; HC, Healthy Control; HDRS, Hamilton Depression Rating Scale; MDD, Major Depressive Disorder; MRI, magnetic resonance imaging; SD, Standard Deviation; SSI, Scale for Suicide Ideation.

**Figure 2:**
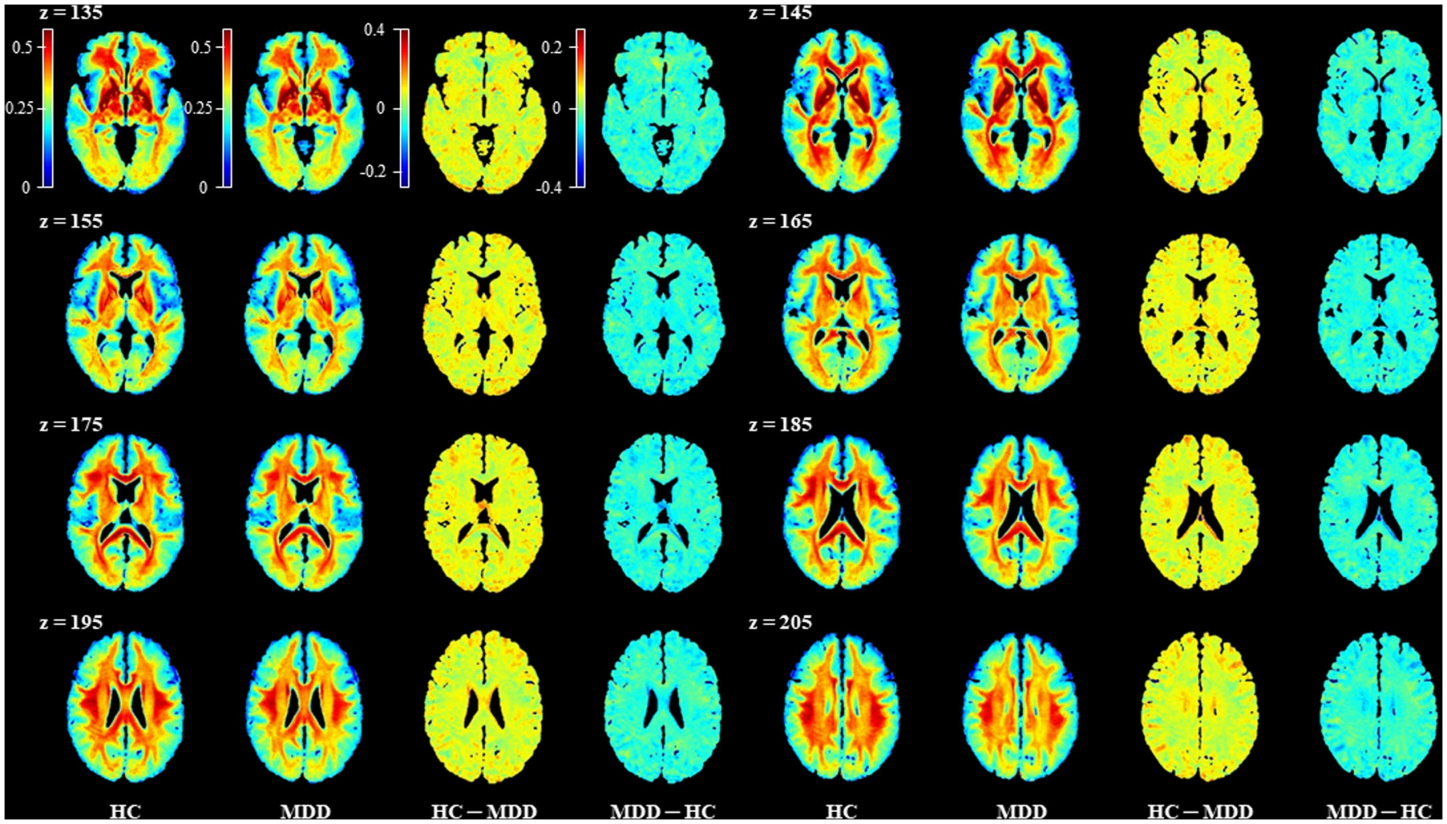
Representative images for comparison of the mean sq-Ratio myelin-related values between the healthy control (HC) and major depressive disorder (MDD) groups. To compare the group average sq-Ratio myelin-related maps on the same scale, each group’s average sq-Ratio myelin-related map was normalized to have values between 0 and 1. When the normalized group average myelin-related maps were subtracted from each other, the HC – MDD myelin-related map had values ranging from −0.2 to 0.4, indicating that the average myelin-related value in the HC group was higher than that in the MDD group.

We compared the sq-Ratio myelin-related values between the HC and MDD groups in the white matter and subcortical regions. The average sq-ratio myelin-related value of the HC group was higher than that of the MDD group in both white matter and subcortical regions. The average sq-Ratio myelin-related value for the entire ROI in the white matter was 2.62% higher in HCs than in MDD patients (HC, 23.298 ± 1.374 [mean ± standard deviation]; MDD, 22.703 ± 1.480; *p* = 0.085). Additionally, in the subcortical regions, the value was 2.26% larger in HCs than in MDD patients (HC, 17.862 ± 0.966; MDD, 17.467 ± 1.280; *p* = 0.148; Tables 2, 3). Higher myelin-related signals of HCs compared to MDD patients were also clear (Figure 2).

**Table 2:** Smoothed quantitative ratio (sq-Ratio) myelin-related values and group analysis results in subcortical regions.

ROI name	HC	MDD	*t*	*p*	*p*-FDR
	Mean ± SD	Mean ± SD			
Hippocampus_L	15.466 ± 1.543	14.812 ± 1.806	1.623	0.109	0.468
Hippocampus_R	16.51 ± 1.533	15.765 ± 2.071	1.702	0.094	0.468
ParaHippocampal_L	16.251 ± 2.037	16.619 ± 2.754	−0.631	0.53	0.782
ParaHippocampal_R	17.253 ± 2.318	16.983 ± 2.805	0.436	0.664	0.782
Amygdala_L	15.326 ± 2.373	14.684 ± 2.902	1.011	0.316	0.700
Amygdala_R	16.271 ± 2.362	15.594 ± 2.204	1.241	0.219	0.584
Caudate_L	22.531 ± 2.56	21.484 ± 2.925	1.59	0.117	0.468
Caudate_R	23.379 ± 2.406	22.059 ± 3.217	1.936	0.058	0.387
Putamen_L	31.824 ± 4.38	30.311 ± 4.175	1.48	0.144	0.480
Putamen_R	31.979 ± 4.065	30.715 ± 4.224	1.274	0.207	0.584
Pallidum_L	44.291 ± 5.608	43.005 ± 5.271	0.99	0.326	0.700
Pallidum_R	44.963 ± 4.992	43.685 ± 5.611	1.004	0.319	0.700
Thalamus_L	27.192 ± 2.534	25.038 ± 2.785	3.378	0.001	**0.040**
Thalamus_R	26.297 ± 2.372	24.372 ± 2.698	3.163	0.002	**0.040**
Cerebelum_Crus1_L	18.947 ± 1.871	19.416 ± 2.811	−0.817	0.417	0.725
Cerebelum_Crus1_R	17.803 ± 1.662	18.424 ± 2.428	−1.243	0.219	0.584
Cerebelum_Crus2_L	20.029 ± 3.066	19.481 ± 2.965	0.759	0.45	0.750
Cerebelum_Crus2_R	19.104 ± 2.513	19.239 ± 3.203	−0.196	0.845	0.867
Cerebelum_3_L	11.596 ± 1.217	11.414 ± 1.442	0.57	0.57	0.782
Cerebelum_3_R	10.541 ± 1.048	10.382 ± 1.374	0.54	0.591	0.782
Cerebelum_4_5_L	12.214 ± 1.024	12.107 ± 1.331	0.375	0.709	0.796
Cerebelum_4_5_R	14.33 ± 1.191	14.13 ± 1.894	0.525	0.602	0.782
Cerebelum_6_L	17.1 ± 1.643	17.536 ± 2.146	−0.949	0.346	0.700
Cerebelum_6_R	17.126 ± 1.496	17.506 ± 2.073	−0.876	0.385	0.700
Cerebelum_7b_L	17.091 ± 2.313	15.948 ± 2.629	1.927	0.058	0.387
Cerebelum_7b_R	17.759 ± 2.355	17.22 ± 2.46	0.935	0.353	0.700
Cerebelum_8_L	17.472 ± 2.083	16.446 ± 1.744	2.239	0.028	0.280
Cerebelum_8_R	16.474 ± 1.454	15.599 ± 1.466	2.504	0.015	0.200
Cerebelum_9_L	13.475 ± 1.149	13.478 ± 1.461	−0.01	0.992	0.992
Cerebelum_9_R	12.619 ± 0.894	12.377 ± 1.327	0.892	0.376	0.700
Cerebelum_10_L	11.567 ± 1.526	11.262 ± 2.363	0.637	0.527	0.782
Cerebelum_10_R	10.423 ± 1.299	10.229 ± 2.121	0.458	0.649	0.782
Vermis_1_2	7.409 ± 0.664	7.5 ± 1.305	−0.365	0.716	0.796
Vermis_3	8.01 ± 0.825	7.907 ± 1.118	0.436	0.665	0.782
Vermis_4_5	8.331 ± 0.862	8.206 ± 1.169	0.504	0.616	0.782
Vermis_6	10.208 ± 1.717	10.985 ± 2.106	−1.686	0.097	0.468
Vermis_7	16.408 ± 3.049	17.684 ± 3.879	−1.525	0.132	0.480
Vermis_8	17.343 ± 2.784	17.554 ± 3.55	−0.276	0.783	0.846
Vermis_9	16.962 ± 2.142	16.828 ± 3.164	0.206	0.838	0.867
Vermis_10	4.606 ± 0.577	4.686 ± 0.769	−0.489	0.627	0.782
HC: Mean sq-Ratio myelin-related values ± SD			17.862 ± 0.966		
MDD: Mean sq-Ratio myelin-related values ± SD			17.467 ± 1.280		

*p*-FDR marked with bold indicate statistically significant differences. Caudate, caudate nucleus; Cerebelum_10, lobule X of cerebellar hemisphere; Cerebelum_3, lobule III of cerebellar hemisphere; Cerebelum_4_5, lobule IV, V of cerebellar hemisphere; Cerebelum_6, lobule VI of cerebellar hemisphere; Cerebelum_7b, lobule VIIB of cerebellar hemisphere; Cerebelum_8, lobule VIII of cerebellar hemisphere; Cerebelum_9, lobule IX of cerebellar hemisphere; Cerebelum_Crus1, crus I of cerebellar hemisphere; Cerebelum_Crus2, crus II of cerebellar hemisphere; HC, healthy control; L, left; MDD, major depressive disorder; Pallidum, lenticular nucleus, pallidum; ParaHippocampal, parahippocampal gyrus; Putamen, lenticular nucleus, putamen; R, right; ROI, region of interest; SD, standard deviation; Vermis_1_2, lobule I, II of vermis; Vermis_3, lobule III of vermis; Vermis_4_5, lobule IV, V of vermis; Vermis_6, lobule VI of vermis; Vermis_7, lobule VII of vermis; Vermis_8, lobule VIII of vermis; Vermis_9, lobule IX of vermis; Vermis_10, lobule X of vermis.

**Table 3:** Smoothed quantitative ratio (sq-Ratio) myelin-related values and group analysis results in white matter regions.

ROI name	HC	MDD	*t*	*p*	*p*-FDR
	Mean ± SD	Mean ± SD			
MCP	21.071 ± 1.955	20.468 ± 2.293	1.18	0.242	0.646
PCT	15.1 ± 1.802	15.52 ± 2.399	−0.824	0.413	0.646
GCC	26.389 ± 2.277	25.929 ± 2.241	0.851	0.398	0.646
BCC	26.941 ± 3.091	25.593 ± 4.92	1.363	0.178	0.525
SCC	29.783 ± 2.296	29.094 ± 3.582	0.952	0.345	0.646
FX	19.752 ± 3.39	16.277 ± 4.018	3.9	<0.001	0.012
CST R	11.114 ± 1.517	11.335 ± 2.225	−0.484	0.63	0.742
CST L	10.664 ± 1.635	10.797 ± 2.041	−0.299	0.766	0.851
ML R	11.288 ± 1.528	11.442 ± 2.116	−0.349	0.729	0.828
ML L	16.202 ± 1.957	16.328 ± 2.748	−0.221	0.826	0.878
ICP R	10.459 ± 0.975	10.456 ± 1.365	0.008	0.993	0.993
ICP L	11.772 ± 1.024	11.618 ± 1.6	0.478	0.634	0.742
SCP R	9.107 ± 0.878	9.357 ± 1.267	−0.954	0.344	0.646
SCP L	11.322 ± 1.053	11.546 ± 1.346	−0.773	0.443	0.670
CP R	15.004 ± 2.72	15.665 ± 3.511	−0.877	0.384	0.646
CP L	13.335 ± 2.307	13.711 ± 2.68	−0.628	0.532	0.739
ALIC R	35.934 ± 3.254	34.642 ± 2.606	1.839	0.07	0.380
ALIC L	35.383 ± 2.98	34.314 ± 2.345	1.674	0.099	0.380
PLIC R	31.261 ± 2.072	30.146 ± 3.125	1.749	0.086	0.380
PLIC L	31.102 ± 2.19	30.185 ± 3.037	1.442	0.154	0.496
RLIC R	30.712 ± 2.487	29.552 ± 3.192	1.689	0.096	0.380
RLIC L	32.078 ± 2.432	31.163 ± 2.903	1.426	0.159	0.496
ACR R	31.241 ± 2.637	31.094 ± 2.823	0.224	0.823	0.878
ACR L	29.405 ± 2.641	28.764 ± 2.734	0.997	0.323	0.646
SCR R	30.413 ± 1.959	29.181 ± 3.119	1.966	0.054	0.364
SCR L	29.283 ± 2.019	28.641 ± 2.949	1.057	0.295	0.646
PCR R	28.753 ± 2.276	27.998 ± 3.953	0.971	0.336	0.646
PCR L	27.936 ± 2.116	27.508 ± 4.286	0.525	0.602	0.742
PTR R	31.816 ± 3.337	29.438 ± 3.944	2.716	0.008	0.187
PTR L	31.728 ± 2.391	30.03 ± 3.65	2.288	0.026	0.216
SS R	21.461 ± 2.195	20.902 ± 3.141	0.859	0.394	0.646
SS L	20.895 ± 2.509	21.328 ± 2.752	−0.687	0.495	0.708
EC R	30.069 ± 3.319	28.727 ± 3.317	1.691	0.095	0.380
EC L	25.699 ± 2.436	24.388 ± 2.368	2.283	0.026	0.216
CGC R	28.506 ± 2.552	28.057 ± 1.593	0.888	0.378	0.646
CGC L	32.459 ± 2.218	32.345 ± 2.6	0.198	0.844	0.879
PHC R	17.611 ± 2.471	17.876 ± 2.083	−0.486	0.629	0.742
PHC L	16.928 ± 2.553	16.974 ± 2.483	−0.075	0.94	0.959
FX-ST R	24.388 ± 2.384	23.923 ± 3.202	0.686	0.495	0.708
FX-ST L	23.174 ± 2.239	22.284 ± 2.501	1.566	0.122	0.436
SLF R	33.176 ± 1.971	32.662 ± 2.836	0.876	0.385	0.646
SLF L	32.691 ± 1.916	32.388 ± 2.68	0.542	0.59	0.742
SFO R	33.393 ± 3.154	31.281 ± 3.958	2.46	0.017	0.208
SFO L	33.653 ± 2.78	33.045 ± 3.322	0.828	0.411	0.646
IFO R	26.981 ± 2.615	26.41 ± 2.849	0.873	0.386	0.646
IFO L	23.708 ± 2.179	23.432 ± 2.681	0.472	0.638	0.742
UF R	18.183 ± 2.36	16.642 ± 2.571	2.609	0.011	0.187
UF L	13.914 ± 2.26	14.182 ± 2.087	−0.515	0.608	0.742
Tapetum R	7.849 ± 1.341	6.922 ± 2.465	1.938	0.058	0.364
Tapetum L	3.823 ± 0.588	3.595 ± 1.225	0.986	0.329	0.646
HC: Mean sq-Ratio myelin-related values ± SD			23.298 ± 1.374		
MDD: Mean sq-Ratio myelin-related values ± SD			22.703 ± 1.480		

*p*-FDR marked with bold indicate statistically significant differences. ACR, anterior corona radiata; ALIC, anterior limb of internal capsule; BCC, body of corpus callosum; CGC, cingulum (cingulate gyrus); CP, cerebral peduncle; CST, corticospinal tract; EC, external capsule; FX, fornix (column and body of fornix); FX-ST, fornix (cres)/stria terminalis; GCC, genu of corpus callosum; HC, healthy control; ICP, inferior cerebellar peduncle; IFO, inferior fronto-occipital fasciculus; L, left; MCP, middle cerebellar peduncle; MDD, major depressive disorder; ML, medial lemniscus; PCR, posterior corona radiata; PCT, pontine crossing tract (a part of MCP); PHC, cingulum (hippocampus); PLIC, posterior limb of internal capsule; PTR, posterior thalamic radiation (including optic radiation); R,: right; RLIC, retrolenticular part of internal capsule; ROI, region of interest; SCC, splenium of corpus callosum; SCP, superior cerebellar peduncle; SCR, superior corona radiata; SD, standard deviation; SFO, superior fronto-occipital fasciculus; SLF, superior longitudinal fasciculus; SS, sagittal stratum (including inferior longitudinal fasciculus and IFO); UF, uncinate fasciculus.

In the group analysis between HC and MDD, the sq-Ratio myelin-related values were significantly different in the fornix (FX) area of the white matter (FDR-corrected *p* = 0.012; HC, 19.752 ± 3.39; MDD, 16.277 ± 4.018; Table 2). A significant difference was observed in both the left and right thalamus areas of the subcortical regions (FDR-corrected *p* = 0.040; left thalamus: HC, 27.192 ± 2.534; MDD, 25.038 ± 2.785; FDR-corrected *p* = 0.040; right thalamus: HC, 26.297 ± 2.372; MDD, 24.372 ± 2.698; Table 3). An additional analysis comparing medication status within the MDD group showed no significant differences in each ROI (Supplementary Tables S1, S2). Moreover, in the MDD group, the number of suicide attempts showed significant positive correlations with the mean of sq-Ratio myelin-related values in the subcortex (*r* = 0.491, *p* = 0.004) and white matter (*r* = 0.415, *p* = 0.018; Supplementary Table S3).

## 4. Discussion

The average sq-Ratio myelin-related value of the HC group was higher than that of the MDD group in both the white matter and subcortical regions. In addition, the myelin-related sq-Ratio values were higher in the HC group than in the MDD group in the fornix areas of the white matter and both thalami.

Hou et al. compared myelin in patients with recurrent MDD and HCs using the inhomogeneous magnetization transfer technique developed through the myelin imaging method and showed lower quantitative inhomogeneous magnetization transfer values in the fornix in patients with recurrent MDD (Hou et al., 2021). Additionally, in patients with recurrent MDD, quantitative inhomogeneous magnetization transfer values in the fornix and quantitative myelin transfer values were negatively correlated with disease duration, indicating a decrease in myelin levels as the disease progressed (Hou et al., 2021). Geng et al. also found white matter abnormalities in the fornix, which connects the prefrontal cortex and hippocampus, in patients with early onset MDD, using a resting-state fMRI study (Geng et al., 2016). In a previous DTI study, Li et al. reported white matter disruptions in the fornix and the hippocampal cingulum during late-life depression (Li et al., 2014). The fornix is a part of the limbic system and is a C-shaped bundle of nerve fibers in the brain, which is comprised mostly of efferent fibers from the hippocampus (Griffiths et al., 2009). Hippocampal fibers project *via* the fornix to the orbitofrontal cortex and anterior cingulate, ventral striatum, septal, and preoptic nuclei. Less prominent connections to the anterior thalamic nucleus and hypothalamus also exist (Griffiths et al., 2009). The fornix is a channel for important neurotransmitters such as GABA and acetylcholine, and electrical activity such as theta rhythms in the hippocampus, septal nuclei, Broca’s band, and deep brain structures (Rawlins et al., 1979; Cassel et al., 1997; Hasselmo, 1999). Therefore, the reduction in myelin in depression patients observed in this study may be evidence of the pathophysiology of MDD by impairing connections between brain regions such as the hippocampus and prefrontal cortex, which are important for depression and mental functioning. Our study also showed reduced myelin levels in the thalamus of patients with MDD. To the best of our knowledge, there have been no reports of quantitative abnormalities of thalamic myelin in patients with depression. However, previous studies have provided indirect evidence of reduced myelin levels in the thalamus of patients with depression. Zhang et al. (2021) reported a larger T1 in the left thalamus in patients with MDD and insisted that this finding could be related to abnormal development of the thalamus, such as microstructural proliferation and myelination (Zhang et al., 2022). Jiang et al. reported higher serum levels of myelin oligodendrocyte glycoprotein and myelin-associated glycoprotein, which are related to demyelination in patients with MDD, and decreased fractional anisotropy and axial diffusivity in the white matter of the bilateral thalamus (Jiang et al., 2018). They reported an association between an increase in oligodendrocyte glycoprotein and myelin-associated glycoprotein levels and a decrease in myelin levels in brain regions such as the thalamus (Jiang et al., 2018). The thalamus is a key node in the limbic–cortical-striatal-pallidal-thalamic circuit (Drevets et al., 1992). The thalamus is anatomically interconnected with the prefrontal cortex, striatum, and amygdala, and its reciprocal connections with cortical and subcortical regions facilitate the exchange of subcortical information with the cortex (Price and Drevets, 2010). Previous neuroimaging findings have shown thalamic involvement in the macroscopic structural abnormalities associated with depression. Specifically, diffusion tensor imaging (DTI) studies have shown abnormal structural connectivity of the white matter within the thalamofrontal pathway in MDD patients (Jia et al., 2014; Korgaonkar et al., 2014; Long et al., 2015; Myung et al., 2016).

Previous studies comparing patients with MDD with controls by quantifying myelin have found reduced myelin at the whole-brain level, nucleus accumbens, fornix, left anterior limb of the internal capsule, and left sagittal striatum in patients with MDD (Sacchet and Gotlib, 2017; Hou et al., 2021). Postmortem studies on human brains with depression also showed lower intensity of myelin staining in the dorsolateral prefrontal cortex regions in MDD patients and unipolar and bipolar affective disorders (Regenold et al., 2007; Lake et al., 2017). Although the brain regions showing differences between groups differed among studies, most have shown decreased myelin levels in MDD. There are several hypotheses regarding myelin-related changes in depression, but it seems that stress causes structural alterations in myelin, which in turn may trigger depression (Smaga, 2022). It is possible that the chronic stress associated with depression contributes to decreased myelin levels in the thalamus. Previous mouse experiments have found that chronic social defeat stress causes downregulation of myelin-related genes and is associated with an altered myelin structure (Lehmann et al., 2017), which has been reported to cause depression-like behavior (Birey et al., 2015). Oligodendroglia defects that causes myelin disruption and proposed mechanisms associated with depression are increased levels of circulating corticosterone due to overactivation of HPA axis in stress, pro-inflammatory cytokines and reactive oxygen species released by activated microglia, and epigenetic factors including histone/DNA modification and microRNA (Lutz et al., 2017; Zhang L. et al., 2018; Boda, 2021). Consistent with this mechanism, previous studies suggest that antidepressant use in depressed patients might promote the repair of myelin in the brain (Haroutunian et al., 2014; Boda, 2021). In addition, there are studies showing a correlation between myelin stability, microglial phagocytosis, and synaptic and plasticity-associated proteins with microarray investigation of myelin in relation to suicide, similar to our findings that correlated the number of suicide attempts with sq-ratio values in the MDD group (Klempan et al., 2009; Zhang et al., 2021).

In this study, a reduced myelin concentration in the white matter and subcortical areas of MDD patients was revealed using a more accurate and faster myelin quantification technique in high-resolution 7 T MRI. The sq-Ratio method used in this study enhances contrast by dividing R1 and T2* to detect the presence of myelin, but its limitation is that it can be affected by iron content. Further research on iron content using T2* or quantitative susceptibility mapping is necessary. By incorporating iron content information obtained through such research as a covariate, it is expected that a more precise analysis of myelin can be conducted in terms of sq-Ratio myelin-related values. Although there are many studies on decreased myelin levels in MDD, it is difficult to conclude that myelin reduction in MDD has been clearly demonstrated in the human brain. In the future, we look forward to replicating our results in other populations with larger sample sizes, and future studies on the mechanisms underlying myelin reduction in depression are also warranted. In addition, repeated studies in larger groups are needed to refine the relationship between psychiatric drugs, including antidepressants, and clinical variables such as suicide attempts, duration of illness, and severity of depression.

## Data availability statement

The original contributions presented in the study are included in the article/Supplementary material, further inquiries can be directed to the corresponding authors.

## Ethics statement

The studies involving human participants were reviewed and approved by the study was approved by the Institutional Review Board of Gil Medical Center (IRB no. GDIRB2018-005 and GDIRB2020-207). The patients/participants provided their written informed consent to participate in this study.

## Author contributions

J-MS, S-GK, and C-KK conceived and designed the study. S-EC and S-GK recruited participants and conducted the experiments. J-MS, S-EC, C-KK, and S-GK analyzed the data and wrote the first draft of the manuscript and edited the manuscript. All authors contributed to the article and approved the submitted version.

## Funding

This work was supported by the National Research Foundation of Korea (NRF) grants funded by the South Korean government (MSIT; NRF-2020R1A2C1004355 and NRF-2020R1A2C1007527). This research was funded by the Korea Health Technology R&D Project through the Korea Health Industry Development Institute, which is funded by the Ministry of Health and Welfare, Republic of Korea (grant number HI17C2665).

## Conflict of interest

The authors declare that the research was conducted in the absence of any commercial or financial relationships that could be construed as a potential conflict of interest.

## Publisher’s note

All claims expressed in this article are solely those of the authors and do not necessarily represent those of their affiliated organizations, or those of the publisher, the editors and the reviewers. Any product that may be evaluated in this article, or claim that may be made by its manufacturer, is not guaranteed or endorsed by the publisher.

## Footnotes

^1^ https://github.com/mjt320/T2Star

## Abbreviations

BDI: beck depression inventory
CNS: central nervous system
DTI: diffusion tensor imaging
HC: healthy control
MDD: major depressive disorder
MRI: magnetic resonance imaging
ROI: regions of interest
SPM: statistical parametric mapping
SSI: scale for suicide ideation
