# Supplementary material for: Low myelin-related values in the fornix and thalamus of 7 Tesla MRI of major depressive disorder patients
Source: Front Mol Neurosci. 2023 Aug 10;16:1214738. doi: 10.3389/fnmol.2023.1214738 (PMC10447971; doi:10.3389/fnmol.2023.1214738)
Supplement: Supplementary file 1 [file Data_Sheet_1.docx]

**Supplementary Table 1.** Smoothed quantitative ratio (sq-Ratio) myelin-related values and comparisons by medication status in the MDD group in the subcortical regions.

| **ROI Name** | **Medicated** | **Unmedicated** | **t** | **p** | **p-FDR** |
| --- | --- | --- | --- | --- | --- |
|  | **Mean ± SD** | **Mean ± SD** |  |  |  |
| Hippocampus_L | 14.899 ± 1.934 | 14.407 ± 1.047 | 0.599 | 0.553 | 0.993 |
| Hippocampus_R | 15.911 ± 2.175 | 15.087 ± 1.447 | 0.881 | 0.385 | 0.993 |
| ParaHippocampal_L | 16.975 ± 2.893 | 14.954 ± 0.902 | 3.066 | 0.005 | 0.1 |
| ParaHippocampal_R | 17.293 ± 2.893 | 15.541 ± 1.923 | 1.409 | 0.169 | 0.993 |
| Amygdala_L | 14.836 ± 3.013 | 13.973 ± 2.412 | 0.655 | 0.517 | 0.993 |
| Amygdala_R | 15.693 ± 2.299 | 15.131 ± 1.792 | 0.560 | 0.579 | 0.993 |
| Caudate_L | 21.719 ± 3.073 | 20.385 ± 1.872 | 1.015 | 0.318 | 0.993 |
| Caudate_R | 22.260 ± 3.362 | 21.117 ± 2.445 | 0.786 | 0.438 | 0.993 |
| Putamen_L | 30.863 ± 4.369 | 27.732 ± 1.496 | 3.049 | 0.005 | 0.1 |
| Putamen_R | 31.043 ± 4.551 | 29.186 ± 1.598 | 1.720 | 0.098 | 0.98 |
| Pallidum_L | 42.903 ± 4.563 | 43.480 ± 8.402 | -0.240 | 0.812 | 0.993 |
| Pallidum_R | 43.689 ± 4.930 | 43.667 ± 8.751 | 0.009 | 0.993 | 0.993 |
| Thalamus_L | 24.659 ± 2.852 | 26.808 ± 1.645 | -1.770 | 0.086 | 0.98 |
| Thalamus_R | 24.040 ± 2.810 | 25.921 ± 1.372 | -1.585 | 0.123 | 0.984 |
| Cerebelum_Crus1_L | 19.431 ± 2.875 | 19.343 ± 2.736 | 0.068 | 0.946 | 0.993 |
| Cerebelum_Crus1_R | 18.499 ± 2.593 | 18.076 ± 1.562 | 0.382 | 0.705 | 0.993 |
| Cerebelum_Crus2_L | 19.670 ± 3.140 | 18.606 ± 1.919 | 0.792 | 0.434 | 0.993 |
| Cerebelum_Crus2_R | 19.451 ± 3.457 | 18.247 ± 1.331 | 0.832 | 0.412 | 0.993 |
| Cerebelum_3_L | 11.297 ± 1.224 | 11.961 ± 1.530 | -1.025 | 0.313 | 0.993 |
| Cerebelum_3_R | 10.431 ± 1.465 | 10.158 ± 0.895 | 0.436 | 0.666 | 0.993 |
| Cerebelum_4_5_L | 12.127 ± 1.365 | 12.015 ± 1.272 | 0.185 | 0.855 | 0.993 |
| Cerebelum_4_5_R | 14.185 ± 1.977 | 13.874 ± 1.578 | 0.361 | 0.721 | 0.993 |
| Cerebelum_6_L | 17.719 ± 2.292 | 16.681 ± 0.978 | 1.078 | 0.289 | 0.993 |
| Cerebelum_6_R | 17.629 ± 2.202 | 16.930 ± 1.300 | 0.745 | 0.462 | 0.993 |
| Cerebelum_7b_L | 15.916 ± 2.631 | 16.097 ± 2.869 | -0.150 | 0.881 | 0.993 |
| Cerebelum_7b_R | 17.273 ± 2.526 | 16.976 ± 2.328 | 0.265 | 0.793 | 0.993 |
| Cerebelum_8_L | 16.460 ± 1.787 | 16.378 ± 1.685 | 0.103 | 0.919 | 0.993 |
| Cerebelum_8_R | 15.536 ± 1.464 | 15.895 ± 1.575 | -0.540 | 0.593 | 0.993 |
| Cerebelum_9_L | 13.568 ± 1.523 | 13.057 ± 1.139 | 0.772 | 0.446 | 0.993 |
| Cerebelum_9_R | 12.372 ± 1.373 | 12.397 ± 1.206 | -0.042 | 0.967 | 0.993 |
| Cerebelum_10_L | 11.315 ± 2.559 | 11.016 ± 1.193 | 0.277 | 0.784 | 0.993 |
| Cerebelum_10_R | 10.249 ± 2.262 | 10.135 ± 1.436 | 0.118 | 0.907 | 0.993 |
| Vermis_1_2 | 7.578 ± 1.267 | 7.138 ± 1.545 | 0.744 | 0.462 | 0.993 |
| Vermis_3 | 7.922 ± 1.154 | 7.837 ± 1.027 | 0.167 | 0.868 | 0.993 |
| Vermis_4_5 | 8.206 ± 1.222 | 8.210 ± 0.982 | -0.009 | 0.993 | 0.993 |
| Vermis_6 | 10.996 ± 2.288 | 10.933 ± 0.999 | 0.066 | 0.948 | 0.993 |
| Vermis_7 | 17.893 ± 4.136 | 16.712 ± 2.356 | 0.671 | 0.507 | 0.993 |
| Vermis_8 | 17.774 ± 3.723 | 16.529 ± 2.599 | 0.775 | 0.444 | 0.993 |
| Vermis_9 | 16.780 ± 2.912 | 17.054 ± 4.496 | -0.190 | 0.851 | 0.993 |
| Vermis_10 | 4.703 ± 0.692 | 4.606 ± 1.143 | 0.276 | 0.784 | 0.993 |
| Medicated: Mean sq-Ratio myelin-related values ± SD | | | 17.544 ± 1.333 | | |
| Unmedicated: Mean sq-Ratio myelin-related values ± SD | | | 17.106 ± 1.005 | | |

Abbreviations: Caudate, caudate nucleus; Cerebelum_10, lobule X of cerebellar hemisphere; Cerebelum_3, lobule III of cerebellar hemisphere; Cerebelum_4_5, lobule IV, V of cerebellar hemisphere; Cerebelum_6, lobule VI of cerebellar hemisphere; Cerebelum_7b, lobule VIIB of cerebellar hemisphere; Cerebelum_8, lobule VIII of cerebellar hemisphere; Cerebelum_9, lobule IX of cerebellar hemisphere; Cerebelum_Crus1, crus I of cerebellar hemisphere; Cerebelum_Crus2, crus II of cerebellar hemisphere; L, left; MDD, major depressive disorder; Pallidum, lenticular nucleus, pallidum; ParaHippocampal, parahippocampal gyrus; Putamen, lenticular nucleus, putamen; R, right; ROI, region of interest; SD, standard deviation; Vermis_1_2, lobule I, II of vermis; Vermis_3, lobule III of vermis; Vermis_4_5, lobule IV, V of vermis; Vermis_6, lobule VI of vermis; Vermis_7, lobule VII of vermis; Vermis_8, lobule VIII of vermis; Vermis_9, lobule IX of vermis; Vermis_10, lobule X of vermis.

**Supplementary Table 2.** Smoothed quantitative ratio (sq-Ratio) myelin-related values and comparisons by medication status in the MDD group in the white matter regions.

| **ROI Name** | **Medicated** | **Unmedicated** | **t** | **p** | **p-FDR** |
| --- | --- | --- | --- | --- | --- |
|  | **Mean ± SD** | **Mean ± SD** |  |  |  |
| MCP | 20.789 ± 2.236 | 18.971 ± 2.106 | 1.823 | 0.078 | 0.756 |
| PCT | 15.940 ± 2.325 | 13.558 ± 1.777 | 2.355 | 0.025 | 0.756 |
| GCC | 26.045 ± 1.807 | 25.386 ± 3.883 | 0.407 | 0.699 | 0.813 |
| BCC | 25.265 ± 5.072 | 27.125 ± 4.176 | -0.837 | 0.409 | 0.8 |
| SCC | 29.430 ± 3.563 | 27.526 ± 3.543 | 1.189 | 0.243 | 0.756 |
| FX | 16.022 ± 4.290 | 17.467 ± 2.267 | -0.795 | 0.432 | 0.8 |
| CST R | 11.568 ± 2.288 | 10.248 ± 1.642 | 1.334 | 0.192 | 0.756 |
| CST L | 10.946 ± 2.170 | 10.097 ± 1.154 | 0.923 | 0.363 | 0.756 |
| ML R | 11.651 ± 2.178 | 10.468 ± 1.604 | 1.253 | 0.219 | 0.756 |
| ML L | 16.408 ± 2.867 | 15.954 ± 2.290 | 0.362 | 0.719 | 0.817 |
| ICP R | 10.523 ± 1.423 | 10.145 ± 1.106 | 0.610 | 0.546 | 0.813 |
| ICP L | 11.758 ± 1.695 | 10.961 ± 0.858 | 1.112 | 0.274 | 0.756 |
| SCP R | 9.457 ± 1.218 | 8.888 ± 1.504 | 0.999 | 0.326 | 0.756 |
| SCP L | 11.594 ± 1.282 | 11.322 ± 1.734 | 0.443 | 0.660 | 0.813 |
| CP R | 16.015 ± 3.717 | 14.031 ± 1.687 | 2.017 | 0.059 | 0.756 |
| CP L | 14.004 ± 2.768 | 12.342 ± 1.816 | 1.398 | 0.172 | 0.756 |
| ALIC R | 34.771 ± 2.651 | 34.037 ± 2.518 | 0.621 | 0.539 | 0.813 |
| ALIC L | 34.523 ± 1.855 | 33.338 ± 4.041 | 0.703 | 0.511 | 0.813 |
| PLIC R | 29.815 ± 3.113 | 31. 693 ± 2.940 | -1.352 | 0.186 | 0.756 |
| PLIC L | 29.807 ± 3.029 | 31.945 ± 2.610 | -1.602 | 0.119 | 0.756 |
| RLIC R | 29.798 ± 2.878 | 28.406 ± 4.536 | 0.969 | 0.340 | 0.756 |
| RLIC L | 31.266 ± 3.114 | 30.679 ± 1.712 | 0.444 | 0.660 | 0.813 |
| ACR R | 31.172 ± 2.599 | 30.733 ± 3.992 | 0.341 | 0.735 | 0.817 |
| ACR L | 28.851 ± 2.407 | 28.357± 4.220 | 0.397 | 0.694 | 0.813 |
| SCR R | 28.892 ± 3.153 | 30.530 ± 2.803 | -1.174 | 0.249 | 0.756 |
| SCR L | 28.389 ± 3.000 | 29.816 ± 2.616 | -1.078 | 0.289 | 0.756 |
| PCR R | 28.081 ± 4.102 | 27.611 ± 3.470 | 0.261 | 0.796 | 0.854 |
| PCR L | 27.356 ± 4.593 | 28.221 ± 2.566 | -0.443 | 0.661 | 0.813 |
| PTR R | 29.582 ± 3.848 | 28.765 ± 4.693 | 0.455 | 0.652 | 0.813 |
| PTR L | 30.155 ± 3.585 | 29.449 ± 4.249 | 0.424 | 0.674 | 0.813 |
| SS R | 21.277 ± 3.195 | 19.149 ± 2.346 | 1.537 | 0.134 | 0.756 |
| SS L | 21.320 ± 2.745 | 21.362 ± 3.050 | -0.033 | 0.974 | 0.98 |
| EC R | 28.886 ± 3.521 | 27.983 ± 2.205 | 0.599 | 0.553 | 0.813 |
| EC L | 24.529 ± 2.535 | 23.731 ± 1.300 | 0.744 | 0.462 | 0.813 |
| CGC R | 28.106 ± 1.374 | 28.827 ± 2.544 | 0261 | 0.803 | 0.854 |
| CGC L | 32.337 ± 2.311 | 32.381 ± 3.971 | -0.026 | 0.980 | 0.98 |
| PHC R | 18.157 ± 2.048 | 16.570 ± 1.873 | 1.745 | 0.091 | 0.756 |
| PHC L | 17.178 ± 2.594 | 16.018 ± 1.733 | 1.041 | 0.306 | 0.756 |
| FX-ST R | 24.071 ± 3.315 | 23.234 ± 2.764 | 0.575 | 0.569 | 0.813 |
| FX-ST L | 22.309 ± 2.670 | 22.166 ± 1.664 | 0.125 | 0.901 | 0.939 |
| SLF R | 32.507 ± 2.446 | 33.383 ± 4.474 | -0.681 | 0.501 | 0.813 |
| SLF L | 32.212 ± 2.694 | 33.208 ± 2.694 | -0.822 | 0.417 | 0.8 |
| SFO R | 30.956 ± 4.034 | 32.798 ± 3.488 | -1.036 | 0.308 | 0.756 |
| SFO L | 32.798 ± 3.185 | 34.198 ± 4.017 | -0.935 | 0.357 | 0.756 |
| IFO R | 26.828 ± 2.870 | 24.456 ± 1.876 | 1.926 | 0.063 | 0.756 |
| IFO L | 23.753 ± 2.731 | 21.930 ± 1.969 | 1.543 | 0.133 | 0.756 |
| UF R | 16.863 ± 2.651 | 15.612 ± 2.033 | 1.085 | 0.286 | 0.756 |
| UF L | 14.389 ± 2.154 | 13.213 ± 1.527 | 1.264 | 0.215 | 0.756 |
| Tapetum R | 7.018 ± 2.415 | 6.475 ± 2.886 | 0.484 | 0.632 | 0.813 |
| Tapetum L | 3.654 ± 1.173 | 3.319 ± 1.536 | 0.602 | 0.552 | 0.813 |
| Medicated: Mean sq-Ratio myelin-related values ± SD | | | 22.781 ± 1.468 | | |
| Unmedicated: Mean sq-Ratio myelin-related values ± SD | | | 22.342 ± 1.600 | | |

Abbreviations: ACR, anterior corona radiata; ALIC, anterior limb of internal capsule; BCC, body of corpus callosum; CGC, cingulum (cingulate gyrus); CP, cerebral peduncle; CST, corticospinal tract; EC, external capsule; FX, fornix (column and body of fornix); FX-ST, fornix (cres)/stria terminalis; GCC, genu of corpus callosum; ICP, inferior cerebellar peduncle; IFO, inferior fronto-occipital fasciculus; L, left; MCP, middle cerebellar peduncle; MDD, major depressive disorder; ML, medial lemniscus; PCR, posterior corona radiata; PCT, pontine crossing tract (a part of MCP); PHC, cingulum (hippocampus); PLIC, posterior limb of internal capsule; PTR, posterior thalamic radiation (including optic radiation); R,: right; RLIC, retrolenticular part of internal capsule; ROI, region of interest; SCC, splenium of corpus callosum; SCP, superior cerebellar peduncle; SCR, superior corona radiata; SD, standard deviation; SFO, superior fronto-occipital fasciculus; SLF, superior longitudinal fasciculus; SS, sagittal stratum (including inferior longitudinal fasciculus and IFO); UF, uncinate fasciculus.

**Supplementary Table 3. Partial correlation coefficients(r) between** sq-Ratio myelin-related values and clinical variables in the MDD group

|  | CGI-S | | | HDRS-17 | | BDI | | | duration of illness | | | Number of suicide attempts | | |
| --- | --- | --- | --- | --- | --- | --- | --- | --- | --- | --- | --- | --- | --- | --- |
| Regions | r | *p* | r | | *p* | | r | *p* | | r | *p* | | r | *p* |
| subcortex |  |  |  | |  | |  |  | |  |  | |  |  |
| Thalamus_L | -0.096 | 0.601 | 0.108 | | 0.558 | | 0.037 | 0.841 | | 0.030 | 0.871 | | 0.058 | 0.753 |
| Thalamus_R | -0.100 | 0.585 | 0.089 | | 0.628 | | -0.048 | 0.796 | | -0.087 | 0.635 | | -0.001 | 0.995 |
| Average | 0.056 | 0.762 | −0.086 | | 0.640 | | 0.024 | 0.897 | | 0.279 | 0.123 | | **0.491** | **0.004**^*^ |
| white matter |  |  |  | |  | |  |  | |  |  | |  |  |
| Fornix | -0.132 | 0.470 | 0.041 | | 0.825 | | -0.131 | 0.475 | | -0.209 | 0.250 | | 0.341 | 0.056 |
| Average | -0.095 | 0.607 | −0.084 | | 0.646 | | -0.323 | 0.072 | | -0.002 | 0.991 | | **0.415** | **0.018**^*^ |

Adjusted for age and sex

*Significant at p < 0.05

Abbreviations: BDI, Beck Depression Inventory; CGI-S, Clinical Global Impression Severity Scale; HDRS-17, Hamilton Depression Rating Scale 17 items; L, left; MDD, Major Depressive Disorder; R, right.
